# Effect of Sulfur-Containing Primers for Noble Metals on the Bond Strength of Self-Cured Acrylic Resin

**DOI:** 10.3390/dj5020022

**Published:** 2017-06-20

**Authors:** Keiichi Yoshida

**Affiliations:** Clinic of Fixed Prosthodontics, Nagasaki University Hospital, 1-7-1, Sakamoto, Nagasaki 852-8588, Japan; keiichi@nagasaki-u.ac.jp

**Keywords:** bond strength, metal primer, noble metal, sulfur-containing, thermal cycling

## Abstract

This study investigated the effect of sulfur-containing primers for noble metals on the shear bond strength of self-cured acrylic resin after thermal cycling (TC). Four pure metals (Au, Ag, Cu, and Pd) and type IV Au alloy were either untreated, or treated with one of the five sulfur-containing metal primers (V-Primer, Metaltite, Alloy Primer, Metal Link Primer, and Metal Primer Z). Afterwards, a brass ring was placed on the metal surface and filled with self-cured acrylic resin (*n* = 10). The bond strengths were measured after 24 h (TC0) and after 2000 thermal cycles at 4–60 °C (TC2000). Three-way ANOVA and Tukey compromise *post hoc* tests were used to analyze the data (*α* = 0.05). All of the sulfur-containing primers significantly improved the resin bond strength as compared to that of the non-primed group at TC0 regardless of the metal type (*p* < 0.05). However, at TC2000, the bond strengths between the resin and the five metals significantly decreased with respect to the values obtained at TC0 regardless of the primer (*p* < 0.05). The sulfur-containing metal primers, except for Metal Link Primer, were found to be more effective for improving the bond strength between the self-cured acrylic resin and Ag as compared to the other three pure metals (*p* < 0.05). The bond strengths between the resin and Au and type IV Au alloy at TC2000 were the highest ones when Metal Primer Z was used.

## 1. Introduction

Metal crowns and fixed partial dentures (often fabricated from noble metals) are widely used as functional restorations for posterior teeth. Since the strong and durable bonding of resin luting cements to metal surfaces is extremely important for restorations, their adhesion to base metals and enamel substrates can be effectively improved using carboxylic or phosphate promoting monomers [1]. However, these functional monomers were found to be inefficient for noble metal alloys [2]. Therefore, various surface treatment methods have been developed for noble metals, including electrolytic Sn-plating [3,4,5,6,7], high-temperature oxidation [8], the application of a liquid Ga–Sn alloy [9], and silica coating [10]. In addition, sulfur-containing monomers for bonding resins to noble metal alloys were synthesized as alternatives to these techniques [11,12], and marketed as commercially available metal primers. These monomers contain 6-(4-vinylbenzyl-n-propyl) amino-1,3,5-triazine-2,4-dithiol (VBATDT) [13], thiophosphoric acid derivatives (MEPS) [14,15], 6-methacryloyloxyhexyl 2-thiouracil-5-carboxylate (MTU-6) [16], or 10-methacryloyloxydecyl-6,6-dithiooctanate (MDDT) [17], and their single-liquid primers can be easily applied onto noble metal surfaces without special equipment or complicated surface treatment procedures. It has been also reported that the sulfur-containing monomers promoted the adhesion between the resin and pure metals, such as Au, Ag, Cu, and Pd [12,13]. The sulfur-containing monomers contained in commercially available metal primers were effective for improving the bond strength of resin to noble alloys [18,19,20,21]. However, particular types of the sulfur-containing metal primers that result in the most durable bonding between the resin and the metal elements of gold alloys still remain unknown.

The purpose of this study was to determine the most effective sulfur-containing metal primers for improving the bonding durability of self-cured acrylic resin to pure Au, Ag, Cu, and Pd, which represent the elements of type IV gold alloy. To achieve this goal, the bond strength and bonding durability of self-cured acrylic resin to the four pure metals and type IV Au alloy were evaluated using six different sulfur-containing metal primers.

## 2. Materials and Methods

**Figure 1:**
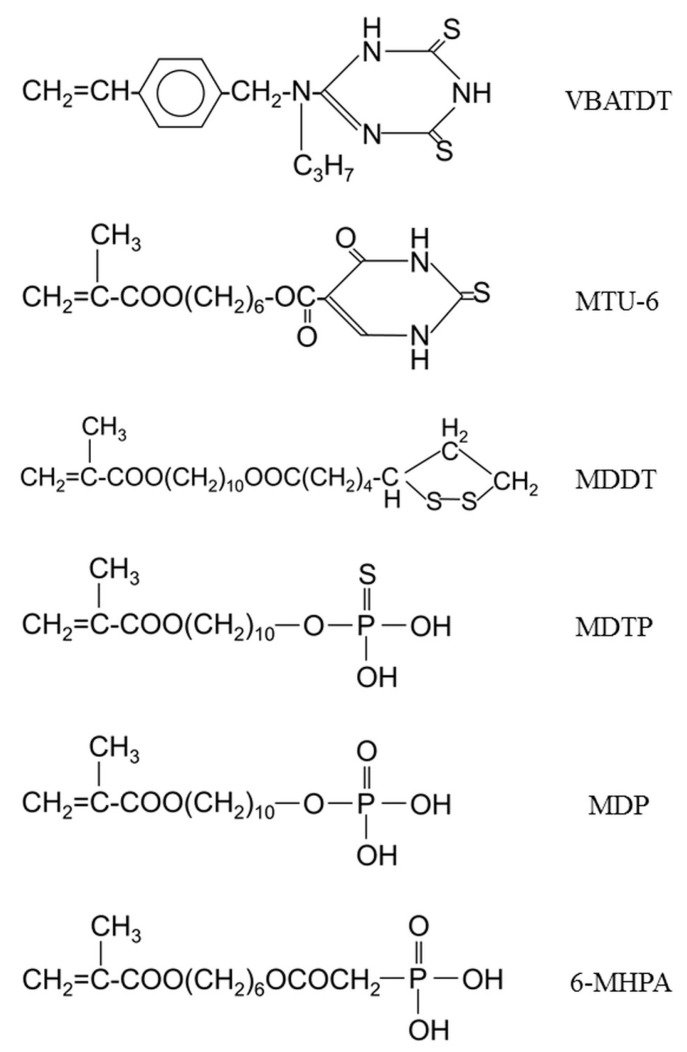
Chemical structures of four sulfur-containing monomers and two phosphoric acid monomers.

The descriptions of the four pure metals and type IV Au alloy, five commercially available sulfur-containing metal primers and one experimental sulfur-containing primer, and self-cured acrylic resin investigated in this study are given in Table 1. The chemical structures of the monomers contained in the metal primers are shown in Figure 1.

### 2.1. Preparation of Bonding Specimens

Disk metal specimens (8.0 mm in diameter, 2.0 mm in thickness) of the four pure metals were supplied by the manufacturer, while the disk specimens of type IV Au alloy with the same dimensions were cast according to the manufacturer’s instructions. The surfaces of all bonding metal specimens were polished with #1000 Si carbide paper (Marumoto Struers Co., Ltd, Tokyo, Japan), followed by ultrasonic cleaning for 10 min in distilled water and subsequent drying with oil-free air for 5 s.

The bonding surfaces were primed with one of the six sulfur-containing metal primers using a sponge microbrush, and then dried with oil-free air for 5 s. Pieces of polyethylene adhesive tape (approximately 50 µm in thickness) with 4.0-mm-diameter circular holes were placed on the pretreated surfaces of the metal specimens to define the bonding area. A brass ring (5 mm inner diameter, 7 mm outer diameter, and 2 mm thickness) was placed on the tape top, and then filled with self-cured acrylic resin using a brush-on technique. The non-primed specimens were also prepared for each of the five metals in the same manner (None). The bonded specimens were stored at a room temperature of 22 ± 2 °C for 30 min.

Each specimen group was divided into two subgroups (*n* = 20), corresponding to two different storage conditions. One subgroup (*n* = 10) was stored in distilled water at 37 °C for 24 h, while the other subgroup (*n* = 10) was subjected to 2000 thermal cycles (TC) between two different water baths (Rika-Kogyo, Hachioji, Japan) maintained at 4 °C and 60 °C; the dwelling time in each bath was 1 min.

### 2.2. Shear Testing Procedure

**Figure 2:**
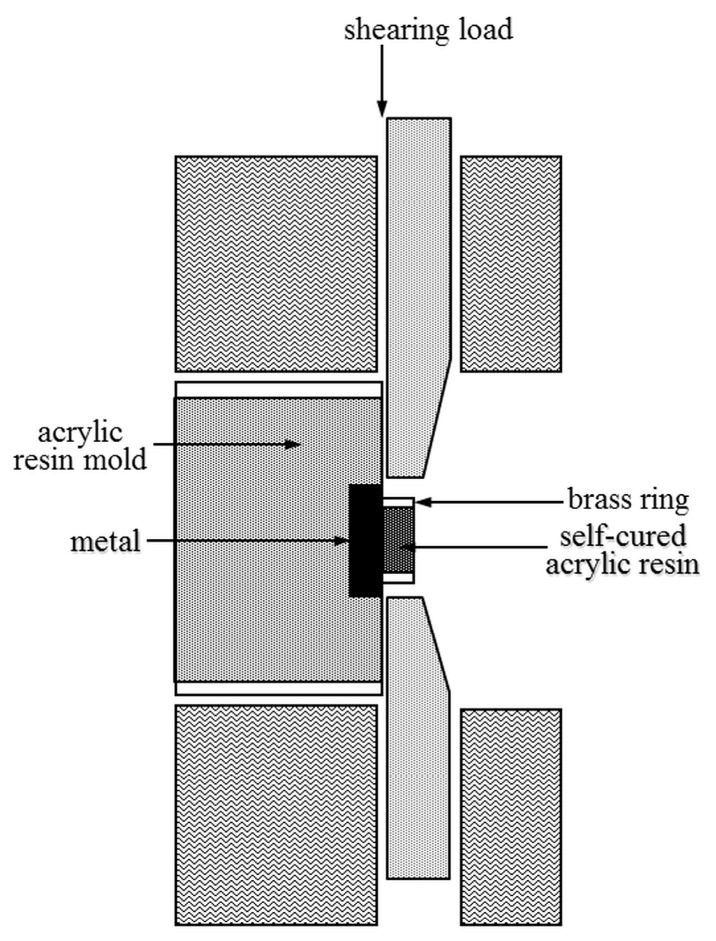
Shear test configuration.

Each bonded specimen was embedded in an acrylic resin mold, and then placed inside an ISO/TR 11405 shear tests jig. The shear bond strengths were measured using a universal testing machine (AGS-10kNG, Shimazu Corp., Kyoto, Japan), with a load applied in the direction parallel to the bonding surface at a crosshead speed of 0.5 mm/min (Figure 2). The shear bond strength was calculated by dividing the force, at which bond failure occurred, by the bonding area.

The debonded surfaces were examined with an optical microscope (SMZ-10, Nikon Corp., Tokyo, Japan) at a magnification of ×45 to evaluate possible failure types. The observed failure modes were classified as either (i) an adhesive failure at the metal–acrylic resin interface, or (ii) a mixed failure of an adhesive failure at the metal–acrylic resin interface and a cohesive failure of the acrylic resin.

### 2.3. Statistical Analysis

SPSS 17.0 (SPSS Inc., Chicago, IL, USA) was used to analyze the results of bond strength measurements. Three-way ANOVA tests were performed for the obtained bond strength values in order to determine the effects produced by the metal types, different metal primers, and thermal cycles. Multiple comparisons of the means were conducted using the *post hoc* Tukey compromise test to identify any significant differences among the groups (*p* < 0.05).

### 2.4. X-Ray Photoelectron Spectroscopy (XPS)

An additional type IV Au alloy specimen was analyzed by XPS measurements, which were performed using a spectrometer (ULVAC-PHI, Inc., 5701LSci, Chigasaki, Japan) equipped with a 2.0 mm × 0.8 mm Al X-ray source. The take-off angle between the specimen surface and the electron optical axis of the spectrometer was maintained at 50°.

## 3. Results

**Figure 3:**
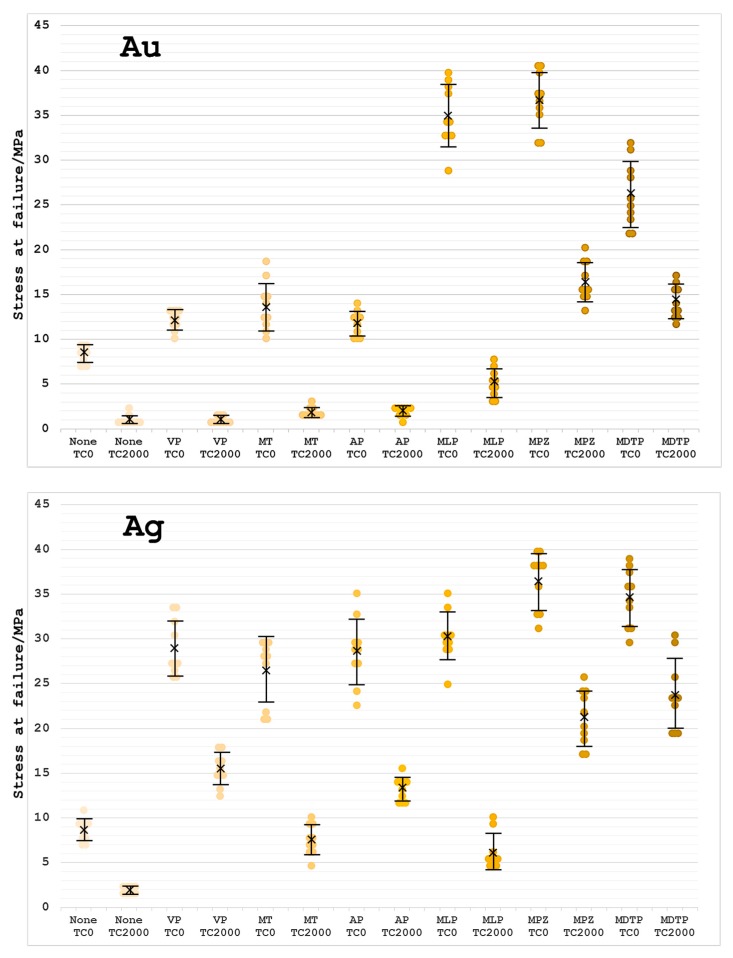

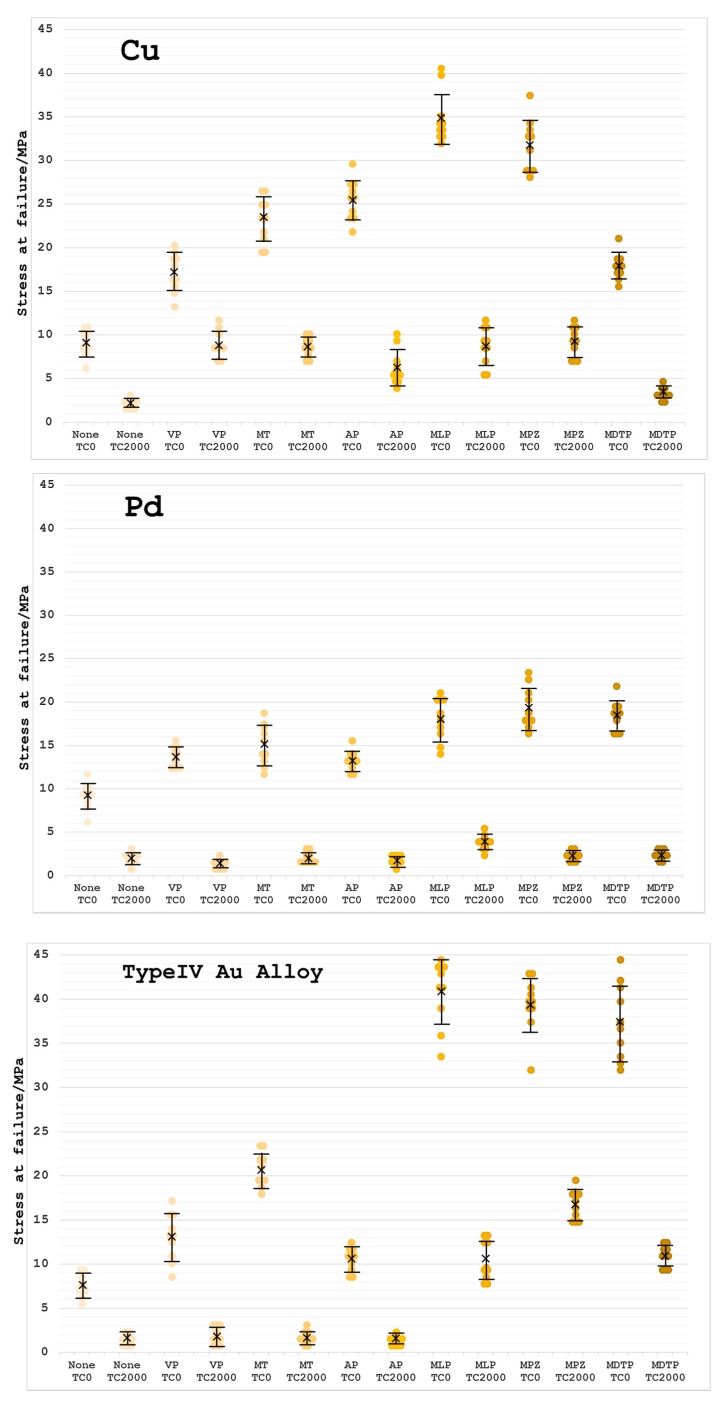
Every raw data point of each shear bond strength present in the scatter diagrams for each metal. The marked × represents the mean value and the error bar standard deviation.

The shear bond strength results failed a test of the homogeneity of variance (*p* < 0.001). However, this was resolved by a square root transformation when sufficient homogeneity was achieved (*p* = 0.0642). The results of three-way ANOVA on the transformation data (Table 2) showed that the effects of the metal primer, metal type, and thermal cycling procedure on the bond strength were statistically significant, and their interactions were also significant (*p* < 0.0001). The mean values, standard deviations in parentheses, and significant differences of the shear bond strengths between the self-cured acrylic resin and five metals obtained at TC0 and TC2000 are listed in Table 3 and graphically presented in Figure 3.

For the non-primed specimens at TC0, the bond strengths between the acrylic resin and pure metals were equal to approximately 8–9 MPa regardless of the metal type. On the other hand, when primed with one of the six metal primers, the bond strengths at TC0 were significantly higher than those obtained for the non-primed group (*p* < 0.05). At TC2000, the bond strengths between the acrylic resin and pure metals significantly decreased as compared to the corresponding values at TC0 regardless of the metal primer (*p* < 0.05). For Au and Ag primed with MPZ or MDTP, the bond strengths of the acrylic resin at TC2000 were significantly higher than the values obtained for the groups primed with the other four primers (*p* < 0.05). For Cu primed with MLP or MPZ, the bond strength at TC0 was more effectively improved among the six primers (*p* < 0.05). For Pd primed with MLP, MPZ, or MDTP, the bond strengths at TC0 were higher than the values obtained for the other three primers (*p* < 0.05). However, the bond strength of the acrylic resin to Pd at TC2000 was not significantly different from the corresponding values obtained for the non-primed group, except for the MLP primer (*p* > 0.05).

When primed with one of the six metal primers, the bond strength between the self-cured acrylic resin and type IV Au alloy was found to be significantly higher than the value obtained for the non-primed group at TC0 (*p* < 0.05). However, at TC2000, the bond strengths were very similar to those obtained for the non-primed group, except for the MLP, MPZ, and MDTP primers (*p* < 0.05).

**Figure 4:**
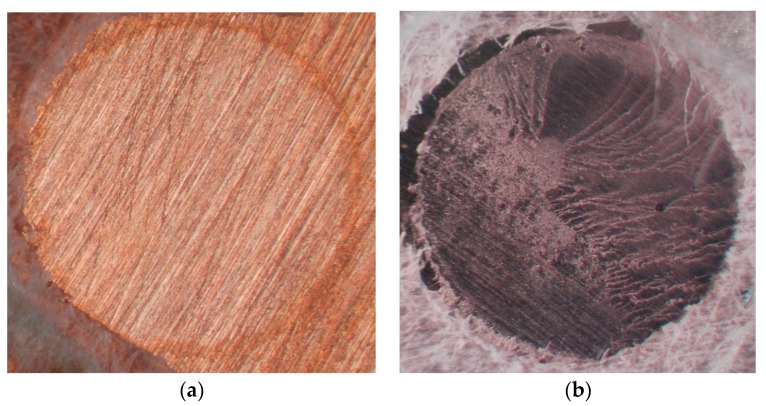
Microscopy image (×45) representative failure. (**a**) adhesive failure at Au–self-cured acrylic resin interface, (**b**) mixed failure of the adhesive failure at Au alloy–self-cured acrylic resin interface and the cohesive failure of self-cured acrylic resin.

Table 4 shows the failure types for the fabricated resin–metal systems. All the non-primed specimens exhibited adhesive failures at the resin–metal interface regardless of the metal type or thermal cycling procedure (Figure 4a). In contrast, no adhesive failures were observed for the Au specimens primed with MPZ or MDTP and for type IV Au alloy primed with MLP, MPZ, or MDTP before and after TC, as indicated by the traces of the self-cured acrylic resin that remained on the metal surface (Figure 4b).

**Figure 5:**
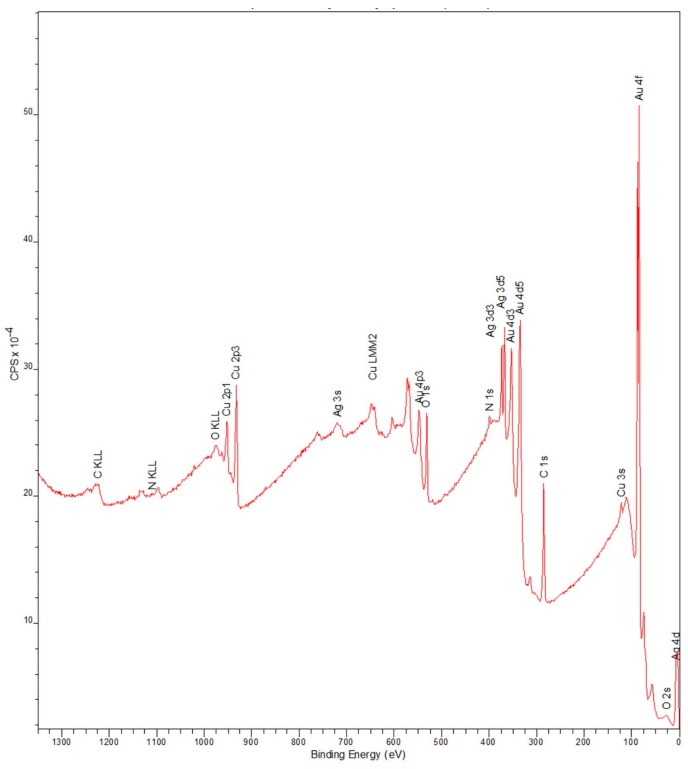
X-ray photoelectron spectroscopy (XPS) spectra for type IV Au alloy.

**Figure 6:**
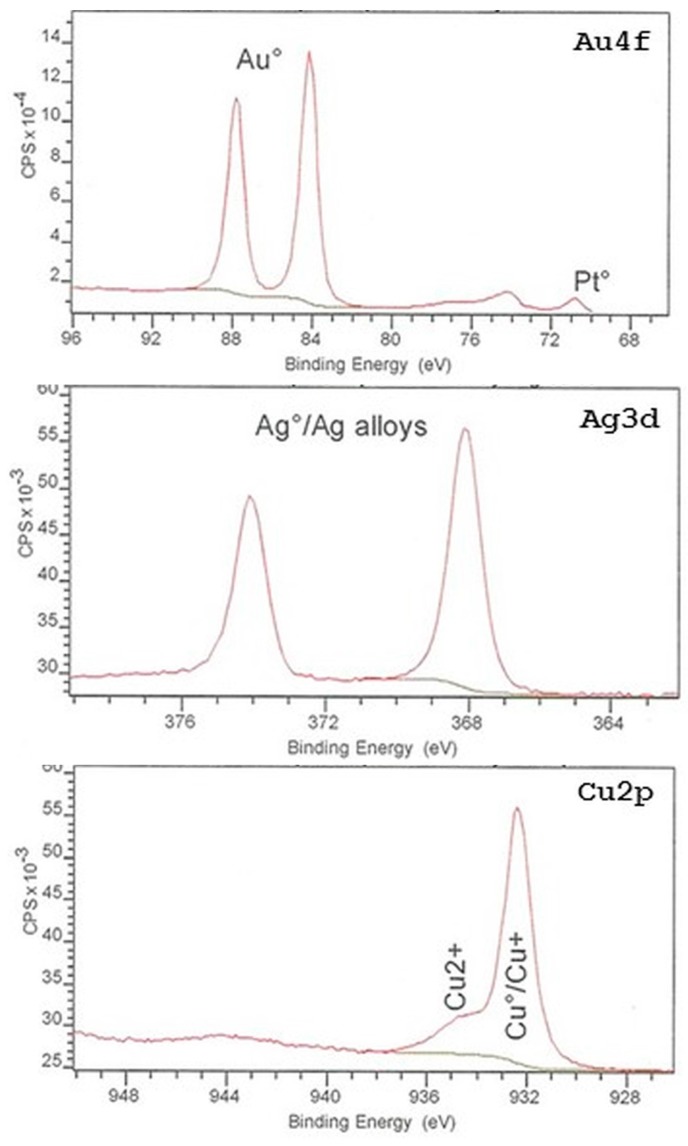
X-ray photoelectron spectroscopy (XPS) spectra of Au4f, Ag3d, and Cu2p for type IV Au alloy.

Figure 5 shows the wide scan XPS spectrum peaks with the energy values corrected with respect to the C 1s peak (284.8 eV). The XPS analysis revealed the presence of Au, Cu, Ag, Pt, O, C, and N elements on the surface of type IV Au alloy. Pd atoms (Pd 3d_5/2_ 335.2 eV) were not detected because their energy peaks were very close to the Au ones (Au 4d_2/5_ 334.8 eV, it was also difficult to detect small amounts of Pd when large amounts of Au were present). According to the obtained XPS binding energies, the Au and Pt (Figure 6, upper panel) as well as Ag (Figure 6, center) elements were present in their metal states, while the Cu element (Figure 6, lower panel) was present both in its metal state (932.7 eV) and as Cu_2_O (932.4 eV, their corresponding binding energies were very close). In addition, small amounts of CuO (933.8 eV) were detected as well.

## 4. Discussion

The bond strengths of the self-cured acrylic resin to the four pure metals primed with one of the six sulfur-containing metal primers increased significantly as compared to the non-primed group at TC0 (*p* < 0.05). However, at TC2000, the bond strengths between the resin and the five metals significantly decreased with respect to the values obtained at TC0 regardless of the primer (*p* < 0.05). The sulfur-containing metal primers, except for Metal Link Primer, were more effective for improving the bond strength between the self-cured acrylic resin and Ag as compared to the other three pure metals (*p* < 0.05).

Shear bond strength testing has been conducted in previous studies because the presence of non-uniform interfacial stress may cause cohesive failures of the bonding substrate and misinterpretation of the obtained data [22]. In addition, the stress concentration near the loading site reduced the calculated value of the shear bond strength below the true failure stress level [23]. In the current study, adhesive failures were mainly observed at TC2000, except for Ag primed with VP, AP, MPZ, or MDTP primer; Au primed with MPZ or MDTP primer; and type IV Au alloy primed with MLP, MPZ, or MDTP primer (no cohesive failures occurred in the self-cured acrylic resin). Hence, it appeared possible to evaluate the effect produced by several metal primes on the bonding of the self-cured resin to noble metals after TC *via* shear testing.

TC was used as a standard procedure for simulating the aging process [13,14,15,16,17,18,19,20,21]. It utilizes the differences in the thermal coefficients of the expansion of the metal and acrylic resin, which induce stress in the adhesive bonding of various functional monomers contained in the utilized primers to the studied metal surfaces. Although it is not possible to fully simulate the environment inside the oral cavity under laboratory conditions, including the moisture and stress induced at the interface between the teeth and the restorations, laboratory conditions can, to some extent, simulate the oral cavity environment through the aging procedure. Among various laboratory processes that are capable of reproducing dynamic stress, TC represents one of the most widely used aging methods, and it is often referenced in the literature [24]. Temperature changes inside the oral cavity are dynamic in nature; thus, it is very hard to define the temperature range corresponding to the mouth physiology. It has been reported previously that the TC temperatures commonly utilized by researchers were too extreme to provide a representative simulation of the temperature fluctuations in vivo [25]. However, the range between 4 °C and 60 °C used in this study was adopted as a reference, which was in partial agreement with the ISO 11405 recommendations [26], and allowed evaluation of the intraoral temperature ranges in vivo [27].

Generally, when metal primers are used clinically, adhesive resin cements containing effective promoting monomers are prepared for luting restorations. When used with Super-Bond C&B as luting cement, its bond strengths to Au, Ag, and Cu without metal primers exceeded 35 MPa [28], which were much higher than the values obtained in our study. Since it is difficult to evaluate the effectiveness of primers for noble metals when adhesive resin cement is used, self-cured acrylic resin without functional monomers was prepared in this study. The internal surfaces of metal restorations are usually alumina-blasted by dental laboratories after casting to remove the reaction layers of the investment material. In this study, the surfaces of the tested metal specimens were only polished with Si carbide paper in order to reduce the mechanical retention of the self-cured acrylic resin as compared to the alumina-blasted specimens, although even the polishing of the metal surface with Si carbide paper was unable to completely eliminate the mechanical retention. Thus, the effectiveness of the utilized metal primers has been evaluated adequately in this work.

VBATDT has been synthesized as a sulfur-containing monomer [11], which significantly enhanced the bond strength and bonding durability of the Ag and Cu surfaces [12,13]. In addition, improved bond strength was also reported for the Pd and Au metals [12,29]. VBATDT is chemisorbed via sulfur atoms on the Au, Ag, and Cu surfaces, assuming a triazine dithiol-type ring structure [30]. In this study, the VBATDT-containing VP and AP primers and the MT primer were more effective for Ag at TC0 and TC2000 as compared to the other four metals. On the other hand, the MLP primer was more effective for Au, Ag, Cu, and type IV alloy at TC0 as compared to Pd; while at TC2000, higher bond strengths were observed for Cu and type IV Au alloy as compared to the values obtained for the other three metals. The MDTP-containing MPZ primer was more effective for Au and Ag than for Cu and Pd at TC0 and TC2000. Thus, MDTP was found to be effective for improving the bond strength of the resin to pure Au and Ag. The sulfur-containing monomers contained in the five commercially available metal primers differed in their chemical structures, which might influence the strength of the chemical bonding to the noble metal surfaces. Therefore, the bonding durability of the self-cured acrylic resin to the noble metals depended on the utilized metal primer brand.

For the pure Au, Ag, Cu, and Pd evaporated onto glass substrates, the Au and Pd elements were present in their metal states, while the Ag and Cu elements were detected by XPS in the forms of Ag_2_O, Cu_2_O, and CuO oxides [31]. In this study, the Au, Ag, Pd, and Pt elements were present on the surface of type IV Au alloy in their metal states, while the Cu element was detected both in its metal state and as small amounts of Cu_2_O and CuO oxides, as determined by XPS (Figure 5 and Figure 6). It was found that the sulfur-containing monomers did not react with the oxide layers that were formed on base metal surfaces in the atmospheric environment [32]. On the other hand, the chemical adsorption of organic sulfur-containing compounds was observed on the Au surface followed by the formation of monolayer films, which resulted in their chemical interaction with the Au surface atoms [30]. Therefore, each sulfur-containing monomer contained in the utilized metal primers might form chemical bonds with predominantly Au metal atoms on the type IV Au alloy surface, thus leading to their adhesion. However, the durability of the chemical bonding with the Au atoms depended on the metal primer brand. Among the studied sulfur-containing monomers, the MPZ primer exhibited the highest bonding durability of the resin to pure Au and type IV Au alloy.

The VBATDT-containing AP primer or the MDTP-containing MPZ primer was more effective for Cu than the VP or MDTP primers, respectively. It should be noted that both the AP and MPZ primers contain MDP in addition to the VBATDT and MDTP monomers, respectively. Moreover, among the six metal primers, the highest bond strength between the resin and Cu at TC0 was obtained with the MLP primer. MLP contains 6-MHPA phosphoric acid monomer, which is very similar to MDP, while both MDP and 6-MHPA exhibit affinity to the oxide layer generated on the metal surface [18,21]. Therefore, both MDP and 6-MHPA may react with the Cu oxide layer that is formed on the surface of pure Cu.

## 5. Conclusions

The sulfur-containing metal primers, except for Metal Link Primer, more effectively improved the bond strength between the self-cured acrylic resin and Ag as compared to Au, Cu, and Pd. The bonding durability depended on the utilized metal primer brand. The MDTP-containing MPZ primer was preferable for type IV Au alloy, because it bonded the self-cured acrylic resin to the predominant Au surface atoms most efficiently.

## Figures and Tables

**Table 1:** Materials used in the study.

Material	Manufacturer	Batch Number	Component	Code
Pure gold	High Purity Chemicals, Itado, Japan	4148021	Au > 99.99	Au
Pure silver	High Purity Chemicals, Itado, Japan	4124692	Ag > 99.99	Ag
Pure copper	High Purity Chemicals, Itado, Japan	4124691	Cu > 99.99	Cu
Pure palladium	High Purity Chemicals, Itado, Japan	4124693	Pd > 99.99	Pd
Casting gold M.C. Type IV	GC Corp., Tokyo, Japan	141071	Au70, Cu16, Ag8, Pd3, Pt2, Zn, Ir1	Type IV Au alloy
V-Primer	Sun Medical, Moriyama, Japan	KT1	VBATDT, acetone	VP
Metaltite	Tokuyama Dental Corp., Kamisu, Japan	03182	MTU-6, ethanol	MT
Alloy Primer	Kuraray Noritake Dental, Tainai, Japan	7E0054	VBATDT, MDP, acetone	AP
Metal Link Primer	Shofu Inc., Kyoto, Japan	101482	MDDT, 6-MHPA, acetone	MLP
Metal Primer Z	GC Corp., Tokyo, Japan	1509101	MDTP, MDP, ethanol	MPZ
MDTP	GC Corp., Tokyo, Japan	1510282	MDTP, ethanol	MDTP
Unifast III	GC Corp., Tokyo, Japan	1506262	Powder: polymethyl methacrylate, ethyl-methyl copolymer, barbituric acid derivative, acethylacetone copper	
		1403041	Liquid: methylmethacrylate, ethyleneglycol dimethacrylate, dimethyl ammonium chloride	

VBATDT: 6-(4-vinylbenzyl-n-propyl) amino-1,3,5-triazine-2,4-dithiol, MTU-6: 6-methacryloyloxyhexyl 2-thiouracil-5-carboxylate; MDP: 10-methacryloyloxydecyl dihydrogen phosphate, MDDT: 10-methacryloyloxydecyl-6,6-dithiooctanate; 6-MHPA: 6-methacryloyloxyhekyl phosphonoacetate, MDTP: 10-methacryloyloxydecyl dihydrogen thiophosphate.

**Table 2:** Three-way ANOVA results for shear bond strength transformed to square root.

Source	Sum of Squares	df	Mean Square	F	*p*
Noble Metal (A)	188.4872	4	47.1218	655.1361	<0.0001
Metal primer (B)	382.1956	6	63.6993	899.1314	<0.0001
Thermal cycling (C)	867.3237	1	867.3237	12,242.4948	<0.0001
A × B	260.1635	24	10.8401	153.0114	<0.0001
A × C	10.3560	4	2.5890	36.5445	<0.0001
B × C	26.1581	6	4.3597	61.5381	<0.0001
A × B × C	32.4949	24	1.3540	19.1114	<0.0001
Error	44.6326	630	0.0708		
Total	1811.8116	699			

**Table 3:** Mean (SD in parenthesis) values of shear bond strength for test groups.

Primer	Metal	Au		Ag		Cu		Pd		Type IV Au Alloy	
	Cycles	0	2000	0	2000	0	2000	0	2000	0	2000
None		8.3	1.0	8.7	2.0	9.1	2.1	9.2	2.0	7.6	1.4
		(1.0) ^a,A^	(0.5) ^a,A^	(1.2) ^a,A^	(0.4) ^a,B^	(1.5) ^a,A^	(0.5) ^a,B^	(1.5) ^a,A^	(0.7) ^ab,B^	(1.3) ^a,A^	(0.6) ^a,AB^
VP		12.2	1.0	28.9	15.4	17.2	8.9	13.7	1.3	13.1	1.9
		(1.1) ^b,A^	(0.4) ^a,A^	(3.1) ^b,C^	(1.8) ^c,C^	(2.2) ^b,B^	(1.6) ^c,B^	(1.2) ^b,A^	(0.5) ^a,A^	(2.7) ^b,A^	(1.0) ^a,A^
MT		13.7	1.9	26.5	7.6	23.2	8.7	15.1	2.0	20.7	1.7
		(2.7) ^b,A^	(0.5) ^a,A^	(3.7) ^b,C^	(1.7) ^b,B^	(2.6) ^c,B^	(1.1) ^c,B^	(2.4) ^b,A^	(0.7) ^ab,A^	(1.9) ^c,B^	(0.7) ^a,A^
AP		11.8	2.0	28.6	13.3	25.5	6.2	13.3	1.7	10.5	1.3
		(1.4) ^b,A^	(0.6) ^a,A^	(3.7) ^b,C^	(1.3) ^c,C^	(2.3) ^c,B^	(2.1) ^b,B^	(1.2) ^b,A^	(0.6) ^ab,A^	(1.3) ^b,A^	(0.5) ^a,A^
MLP		35.0	5.1	30.2	6.2	34.9	8.7	18.1	3.8	41.0	10.4
		(3.5) ^d,C^	(1.6) ^b,AB^	(2.7) ^b,B^	(2.0) ^b,B^	(2.9) ^d,C^	(2.2) ^c,C^	(2.5) ^c,A^	(0.9) ^c,A^	(3.7) ^d,D^	(2.2) ^b,C^
MPZ		36.7	16.5	36.5	21.2	31.7	9.2	19.3	2.3	39.5	16.7
		(3.1) ^d,C^	(2.2) ^c,C^	(3.2) ^c,C^	(3.1) ^d,D^	(3.0) ^d,B^	(1.8) ^c,B^	(2.4) ^c,A^	(0.6) ^b,A^	(3.1) ^d,C^	(1.7) ^c,C^
MDTP		26.2	14.2	34.6	23.7	17.9	3.3	18.4	2.4	37.5	10.9
		(3.7) ^c,B^	(1.9) ^c,C^	(3.2) ^c,C^	(3.9) ^d,D^	(1.5) ^b,A^	(0.7) ^a,A^	(1.7) ^c,A^	(0.6) ^b,A^	(4.3) ^d,C^	(1.2) ^b,B^

Means with the same lowercase superscript letters are not significantly different within the same metal at each thermal cycle (*p* > 0.05); Means with the same uppercase superscript letters are not significantly different within the same primer at each thermal cycle (*p* > 0.05).

**Table 4:** Failure mode distribution in the experimental groups (number of specimens).

Primer	Metal	Au		Ag		Cu		Pd		Type IV Au Alloy	
	Cycles	0	2000	0	2000	0	2000	0	2000	0	2000
None		A10	A10	A10	A10	A10	A10	A10	A10	A10	A10
VP		M10	A10	M10	M10	M10	A10	M10	A10	M10	A10
MT		M10	A10	M10	A10	M10	A10	M10	A10	M10	A10
AP		M10	A10	M10	M10	M10	A10	M10	A10	M10	A10
MLP		M10	A10	M10	A10	M10	A10	M10	A10	M10	M10
MPZ		M10	M10	M10	M10	M10	A10	M10	A10	M10	M10
MDTP		M10	M10	M10	M10	M10	A10	M10	A10	M10	M10

A: adhesive failure at the metal–acrylic resin interface, M: mixed failure of the adhesive failure at the metal–acrylic resin interface and the cohesive failure of acrylic resin.
